# Effect of Previous Cesarean Section on Labor Progression: Comparison Between First VBAC and Primiparous Vaginal Deliveries

**DOI:** 10.3390/jcm14248903

**Published:** 2025-12-16

**Authors:** Maayan Maor, Emmanuel Attali, Eran Ashwal, Omri Dominsky, Yariv Yogev, Yoav Baruch

**Affiliations:** 1 Lis Hospital for Women’s Health, Tel Aviv Sourasky Medical Center, Tel Aviv University, Tel Aviv-Yafo 6423906, Israel; maayan.maor85@gmail.com (M.M.);; 2 Division of Maternal-Fetal Medicine, Department of Obstetrics and Gynecology, McMaster University, Hamilton, ON L8S 4L, Canada

**Keywords:** labor, primiparous, secundiparous, trial of labor after cesarean section, TOLAC, vaginal birth after cesarean section, VBAC

## Abstract

**Background:** The increasing number of cesarean deliveries worldwide has led to a growing population of women eligible for vaginal birth after cesarean (VBAC). limited evidence exists regarding the natural progression of labor among secundiparous women experiencing their first vaginal delivery. Evidence regarding labor progression among women attempting VBAC remains inconclusive and with conflicting results. Clarifying these differences is essential for optimizing intrapartum management. Our objective was to compare the progression rate of the active phase of labor between secundiparous womne at their first VBAC and primiparous women who delivered vaginally. **Methods:** A retrospective cohort study was conducted at a tertiary university-affiliated medical center (January 2011–January 2021). Included were term singleton pregnancies in spontaneous labor resulting in vaginal delivery. Exclusion criteria included induction, augmentation, and operative vaginal delivery. **Results:** Among 13,983 primiparous and 736 VBAC patients, the VBAC group was older, used epidural more frequently, and had higher neonatal birth weight. The cervical dilatation rate during the active phase was faster in VBAC patients (3.26 vs. 2.85 cm/h, *p* = 0.011), with a shorter second stage (77.8 vs. 86.6 min, *p* < 0.001). The rate of prolonged second stage was higher in the primiparous group (9.5% vs. 7.1%, *p* = 0.029). In a multivariable analysis examining the association between VBAC and prolonged second stage, VBAC was found to be inversely associated with prolonged second stage (OR 0.541, 95% CI 0.388–0.753, *p* = 0.001). **Conclusions:** When compared to primiparous women, women at their first VBAC had significantly shorter active phase and increased progression rate as well as a shorter second stage of labor.

## 1. Introduction

Cesarean section (CS) rates have risen steadily worldwide over the past decades, reaching up to 30–35% of all births in many high-income countries, and exceeding 40–50% in several middle-income countries [1]. In a large study including 62,415 women, the most common indication for repeated CS was a patient selection for an elective surgery, accounting for 30.9% of all cesarean deliveries. These findings highlight that reducing primary cesareans and supporting vaginal birth after cesarean (VBAC) are key levers to reduce overall cesarean rates [2].

While maternal morbidity increases progressively with the number of repeated cesarean deliveries, it decreases with the number of successful previous VBAC [3,4].

Consequently, reducing unnecessary repeat cesareans is a clinical and public health priority [5,6,7]. The American College of Obstetricians and Gynecologists (ACOG) practice bulletin has revisited recommendations for VBAC and emphasized the importance of counseling eligible patients about trial of labor after cesarean (TOLAC) [8]. According to the ACOG, most women with one previous low-segment CS are candidates for TOLAC; hence, it should be offered to most women after one CS.

Understanding typical labor patterns when evaluating labor progression rate among secundiparous women attempting TOLAC is clinically important. Misinterpretation of labor curves may prompt unnecessary interventions for presumed protraction [9], whereas recognizing genuinely inadequate progression can support timely action and prevent unnecessary complications.

Contemporary literature estimates VBAC success at 60–80% [10], and even higher in well-selected populations [11]. Despite broad interest in promoting VBAC, data characterizing the physiologic progression of labor, particularly the active phase, during a woman’s first VBAC (i.e., secundiparous women with no prior vaginal birth) remain limited and heterogeneous.

Evidence on labor progression in VBAC versus first vaginal deliveries remains inconclusive.

Several studies found similar active-phase and second-stage patterns to primiparous women, suggesting no need for distinct management [12,13,14,15,16]. In contrast, two smaller studies reported conflicting findings: Lan et al. [17] described a shorter first stage but longer second stage, whereas Li et al. [18] observed the opposite.

This heterogeneity underscores the need for larger, methodologically uniform analyses to define expected labor progression in first VBAC.

Accordingly, we aimed to determine whether labor progression rate differs between secundiparous women at their first VBAC and primiparous women, to better inform clinical expectations and decision-making during labor.

## 2. Materials and Methods

1. **Study design:**

A retrospective cohort study was conducted.

2. **Setting:**

The study was conducted in a tertiary university-affiliated hospital with over 13,000 deliveries annually. Women who had a vaginal delivery between January 2011 and January 2021 were considered for inclusion.

3. **Participants:**

Inclusion criteria included women in their first vaginal delivery, either primiparous women or secundiparous women after CS. Primiparous women were chosen as the comparison group because, similar to secundiparous women undergoing their first VBAC, they had no prior vaginal birth. This allowed for a more homogeneous comparison between groups, eliminating the confounding effect of previous vaginal birth on labor progression.

**Figure 1:**
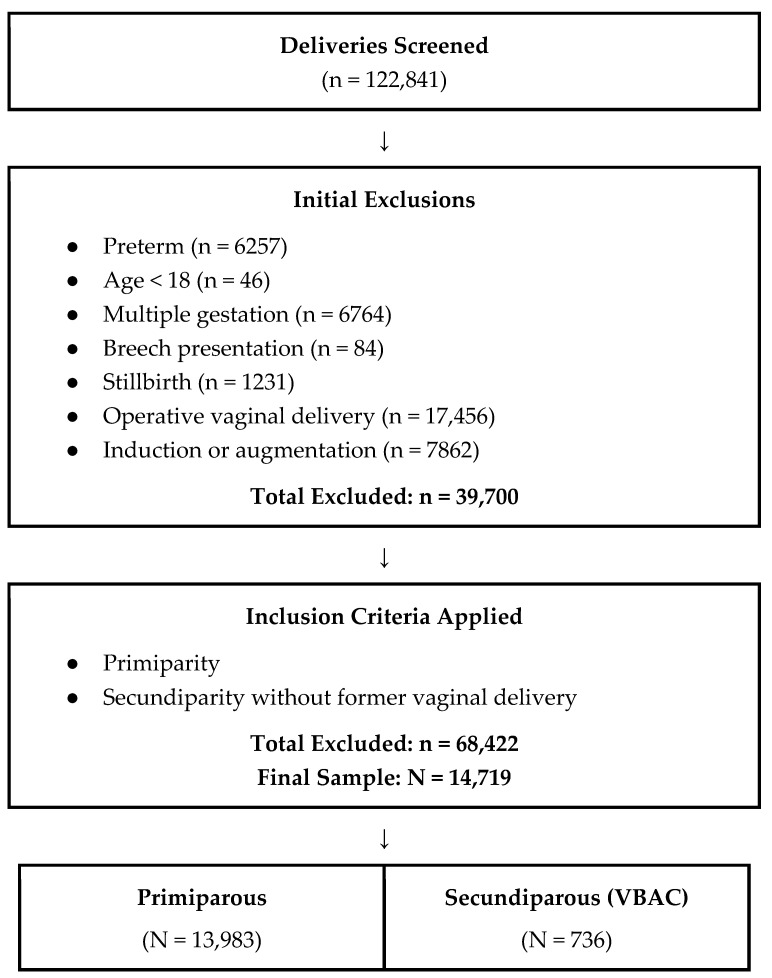
Flowchart of participant selection.

Exclusion criteria included: preterm delivery (before 37 weeks), age < 18, multiple gestation, induction or augmentation of labor, instrumental vaginal delivery, breech presentation at delivery, and stillbirth. A flowchart illustrating patient selection and the study population is presented in Figure 1.

4. **Data collection:**

Variables analyzed included demographic characteristics, medical and obstetric outcomes, intrapartum variables, and neonatal parameters.

A comparison between primiparous women who delivered vaginally and secundiparous women who underwent VBAC was performed. Maternal characteristics included age, height, comorbidities, use of drugs, alcohol, and smoking. Body mass index (BMI) was calculated as weight (kg)/height (m^2^) as measured at the first pregnancy follow-up. Pregnancy and delivery characteristics included parity, gestational age at delivery, gestational diabetes status and weight gain, epidural analgesia, prolonged second stage (PSS), use of episiotomy, and intrapartum fever (defined as a maternal temperature ≥ 38.0 °C).

Fetal and neonatal information included fetal head presentation at birth, sex, weight, and Apgar scores.

Maternal complications covered the degree of perineal lacerations, blood loss, including the need for blood product transfusions. Prolonged second stage was defined as two hours or more (for all women in their first vaginal delivery) with an additional hour for women with epidurals [9].

Labor progression rate in the first stage was calculated as the change in cervical dilatation (cm) divided by the time interval (hours) between examinations.

The beginning of the active phase was set at 3–5 cm dilatation. In case of numerous vaginal examinations revealing cervical dilatation within this range, the most recent measurement was selected.

PSS was defined as ≥2 h for women without epidural analgesia and ≥3 h for women with epidural analgesia, according to ACOG guidelines.

Women who attend our Fetal-Maternal Unit in their second pregnancy, having only one previous low transverse cesarean section, were consulted about the risks of TOLAC on their antenatal visit. They were offered to choose between repeat elective cesarean sections or TOLAC after being carefully explained the advantages and disadvantages of each delivery mode. Women are normally discouraged from attempting TOLAC if a former cesarean was performed due to arrest of dilatation or descent during the active phase of labor, if there is an interval of less than 18 months between deliveries, or if fetal macrosomia is suspected.

5. **Data source**:

Data was extracted from our hospital’s electronic medical records.

6. **Missing Data**:

Missing data was minimal. Variables with missing values included maternal substance use (drugs, smoking, alcohol), likely reflecting incomplete documentation rather than actual prevalence.

7. **Bias**:

To minimize intervention-related bias and accurately assess the natural course of labor, women who underwent any form of medical or instrumental intervention, such as labor augmentation or induction, or operative vaginal delivery, were excluded from the study. This approach aimed to eliminate confounding effects related to altered uterine contractility or assisted delivery on labor progression parameters.

8. **Statistical Analysis:**

Categorical variables were described as frequency and percentage. Continuous variables were evaluated for normal distribution using histograms and quantile-quantile (QQ) plots. Variables that were normally distributed were summarized as mean and standard deviation, while skewed variables were reported as median and interquartile range. Categorical variables were compared between the two groups using the Chi-square test or Fisher’s exact test. Continuous variables and ordinal variables were compared using an independent-samples *t*-test or Mann–Whitney test.

Univariate analysis, as well as multivariable logistic regression, was used to study the association between VBAC and PSS while controlling possible confounders (age, episiotomy, epidural, BMI, duration of second stage, and neonatal weight). All statistical tests were two-sided, and a *p*-value of less than 0.05 was considered statistically significant. SPSS software (IBM SPSS Statistics, version 29, IBM Corporation, Armonk, NY, USA, 2022) was used for all statistical analyses.

9. **Ethical approval**:

The research was conducted in accordance with the principles of the Declaration of Helsinki and institutional ethical standards. The study was approved by the Institutional Review Board (approval no. TLV-0284-08). As this was a retrospective cohort study based on anonymized electronic medical records, informed consent was waived by the committee.

All data were anonymized prior to analysis, and access was restricted to authorized study personnel only.

## 3. Results

Overall, during the study period, 14,719 women were eligible for inclusion in the study population. Of those included, 13,983 (95%) were primiparous women and 736 (5%) were secundiparous women undergoing their first VBAC.

The demographic and obstetrical characteristics of women in both groups are presented in Table 1.

Women in the VBAC group were older (32.6 ± 3.6 vs. 29.71 ± 4.2, *p* < 0.001) and had higher rates of epidural analgesia (634, 86.1% vs. 10,987, 78.6%, *p* < 0.0001). Other than that, the groups were similar regarding demographic and clinical characteristics (Table 1 and Table 2).

The median birth weight in the primiparous group was slightly lower compared to the VBAC group (3234 ± 378 vs. 3266 ± 367 g, *p* = 0.025).

Among women with a previous cesarean section, 105 (33%) underwent an elective cesarean delivery, 194 (61%) underwent a non-elective cesarean delivery prior to the onset of active labor, and 19 (6%) underwent an emergent cesarean delivery. In this study, the terms non-elective and emergent refer to the urgency of the procedure, with non-elective indicating a non-scheduled but non-urgent surgery performed prior to active labor, and emergent indicating a procedure performed under immediate circumstances due to acute maternal or fetal indications.

Indications for the prior cesarean section included 140 (44%) non-vertex presentations, 94 (29.6%) non-reassuring fetal heart rates, 23 (7.2%) arrest of descent/dilatation, 19 (6%) twin pregnancies, 6 (5%) severe preeclampsia, 2 (0.6%) placenta previa, 1 (0.3%) severe IUGR, and 17 (5.3%) other maternal or fetal indications. Notably, only a small proportion of women in the VBAC cohort had a previous cesarean delivery performed due to arrest of dilatation or descent.

The cervical dilatation rate during the active phase of the first stage of labor was significantly faster among VBAC women compared to primiparous women (3.26 vs. 2.85 cm/h, *p* = 0.011).

In contrast, the second stage of labor was significantly shorter among VBAC women (77.8 ± 57.5 min vs. 86.6 ± 59.9 min, *p* < 0.001). The rate of prolonged second stage (PSS) was also lower in the VBAC group compared to primiparous women (7.1% vs. 9.5%, *p* = 0.029).

After the delivery, no difference in the duration of the third stage of birth was noted (15.4 ± 10 min. in the primiparous group vs. 15.1 ± 9.6 in the VBAC group, *p* = 0.386), as well as in the rate of severe perineal lacerations as presented in Table 2.

In a multivariable analysis used to study the association between VBAC and prolonged second stage (Table 3), factors found to be associated with a higher risk for PSS were: maternal age (OR 1.054, CI 95% 1.039–1.071, *p* < 0.001), Caucasian ethnicity (OR 1.828, CI 95% 1.101–3.037, *p* = 0.02), and neonatal birth weight (OR 1.88, CI 95% 1.602–2.204, *p* <0.001).

Factors found to be protective from PSS (Table 3): VBAC (OR 0.541, CI 95% 0.388–0.753, *p* < 0.001) and cervical dilatation rate (OR 0.805, CI 95% 0.771–0.841, *p* < 0.001). In the multivariable regression analysis, epidural analgesia was not significantly associated with a prolonged second stage (OR 1.09, 95% CI 0.31–28.5, *p* = 0.34).

## 4. Discussion

In this current study, we aimed to assess whether secundiparous women who underwent VBAC compared to primiparas have a different labor progression rate during their first vaginal delivery. Our main findings were: (1) women in the VBAC group had a significantly shorter duration of the second stage of birth when compared to primiparous women and were less likely to be diagnosed with PSS. (2) The rate of cervical dilatation (cm/hour) during the active phase of the first stage of labor was significantly faster among women who underwent VBAC.

Women in the VBAC group were older. This age difference can be attributed to the fact that the VBAC delivery was the second pregnancy, reflecting family planning in the study’s population. This may strengthen our results since older maternal age has been associated with prolongation of the second stage of labor [19]. The multiple regression analysis in this study confirmed the association of older maternal age and PSS.

There was a higher rate of epidural analgesia among women in the VBAC group, probably associated with our policy of advising women undergoing TOLAC to use epidural analgesia, which may expedite surgical intervention in case an emergency CS is required [3].

This observation may further strengthen our result as epidural is a known factor associated with a prolonged second stage of labor [20], and the American College of Obstetricians and Gynecologists allotted an additional 1 h for the second stage of labor in women with epidural anesthesia [21].

Miller et al. [22] showed that VBAC women with epidural usually experience a longer second stage of labor when compared with VBAC women without epidural.

In the current study, a multiple regression analysis found that epidural analgesia was not associated with PSS (OR 1.091, *p* = 0.344). The odds ratio (1.09) still trends toward increased risk, so a modest association cannot be excluded in larger samples. Importantly, despite higher epidural rates, women in the VBAC group demonstrated lower rates of prolonged second-stage labor progression, suggesting that the physiologic effect of a second pregnancy may outweigh the expected epidural-related prolongation.

Neonatal weight was found to differ statistically between groups; however, the absolute difference between groups was small and not considered clinically meaningful.

In the multiple regression analysis, neonatal weight was related to a higher risk of PSS (OR 1.88, CI 95% 1.602–2.204, *p* < 0.025) for each additional 1 Kg in birth weight.

Understanding labor patterns among VBAC deliveries may be clinically useful for counseling and guiding clinical management during labor among women attempting a VBAC [3]. Consistent evidence from multiple studies demonstrates that labor duration is longer in primiparous than in multiparous women. Across large contemporary datasets, the median duration has been reported at approximately 10 h versus 6 h, respectively [19,23]. This difference is attributed to reduced soft-tissue resistance and enhanced efficiency of uterine activity with increasing parity [24]. The fact that women in the VBAC group are in practice in their second pregnancy may contribute to their faster labor progression.

Differences in the progression of labor between nulliparous and multiparous women, both in the latent and active phase, have been known for decades, since first described by Friedman [25] in 1955. Since then, considerable changes in both modern obstetric populations and current obstetric management have led to questioning the applicability of Friedman’s nomograms for the progression of labor. Demographic and obstetric factors such as racial diversity, increasing maternal age and BMI, increasing neonatal birthweight, and changes in the rate of regional analgesia are partially responsible for the need for contemporary nomograms of the labor process. As a result, traditional labor curves may no longer apply to contemporary obstetric populations. Since 2010, several studies, evaluated the normal progression of labor in thousands of patients [19,23,26,27].

Up until now, there were only a few studies addressing the progression rate of women during their first VBAC, especially when comparing their delivery’s progression rate to primiparous women. Most current studies showed similar labor progression patterns for both groups, in both the active phase and second stage of the delivery [12,13,14,15,16,28].

Graseck et al. [13] analyzed the active phase of the first stage of labor among over 2800 VBAC women and 4000 primiparous women, demonstrating that their cervical dilatation rates resembled those of primiparous women, suggesting no difference in labor progression. In the study by Faranesh et al. [29], first and second stage labor progression among secundiparous women going through VBAC mirrored that of primiparous women. In contrast, women going through VBAC with a previous vaginal delivery demonstrated a faster progression rate, similar to multiparous women. This study demonstrated that labor progression rate is determined by parity rather than by the mode of delivery.

Although the majority of published studies support similar labor rates and durations, two recent, smaller studies have reported conflicting findings [17,18]. While Li et al. [18] reported faster second-stage progression in the VBAC group, Lan et al. [17] observed opposite results; notably, Lan et al. also described a shorter first stage in the VBAC group.

Rottenstreich et al. reported that a prior cesarean performed in the second stage was associated with shorter labor in subsequent VBAC, whereas cesareans performed electively or during the latent or active first stage were associated with longer labor [30].

In the present study, the main objective was to compare the progression rate as well as the rate of PSS between primiparous and secundiparous women who underwent VBAC. Among women in the VBAC group, progression was significantly faster, and in both univariate and multivariate analyses, VBAC was inversely associated with PSS.

These findings may assist clinicians in counseling eligible women considering trial of labor after cesarean by providing reassurance that, when successful, first VBAC labor progression, particularly during the active and second stages, may be at least comparable and in some aspects even more efficient than that of primiparous women. This may help avoid unnecessary interventions prompted by misinterpretation of labor progression, while maintaining individualized clinical judgment.

10. **Limitations and Strengths:**

The strengths of our study include its relatively large sample size and the long study period in a single tertiary center with a uniform and consistent protocol for TOLAC management. Our strict inclusion criteria, excluding induction, augmentation, and operative vaginal deliveries, allowed us to focus on the natural progression of labor while minimizing potential confounders. Previous studies were limited by smaller or more heterogeneous populations.

Nevertheless, our study has limitations. Its retrospective design inherently restricts causal interpretation. In addition, the findings largely reflect a population of Caucasian women giving birth in a high-volume tertiary center, which may limit generalizability to more diverse or resource-limited settings.

We acknowledge that primiparous women differ from secundiparous women in certain demographic and physiologic characteristics, such as maternal age and parity-related adaptations, which may independently influence labor progression and should be considered when interpreting the results.

Finally, the indication for the prior cesarean and the cervical dilatation at the time of that surgery may have influenced labor progression in the subsequent pregnancy.

Although the indication and timing of the prior cesarean section may influence subsequent labor progression during VBAC, subgroup analyses based on these factors were limited by small sample sizes within specific categories. Further research is needed to delineate potential differences within the VBAC population itself. Specifically, larger cohorts are required to meaningfully compare women who underwent a prior cesarean during active labor with cervical dilatation versus those who had a cesarean section before labor onset.

The available evidence regarding whether the stage of the prior cesarean (elective, first stage, or second stage) influences subsequent VBAC labor progression is controversial. While some reports suggest shorter VBAC duration following a prior in-labor cesarean [31], others have not consistently shown this association [17,18]. In our cohort, although only 33% (n =105) underwent an elective CS, most women who had unplanned cesarean deliveries had their CS prior to any trial of labor. Notably, only a small portion of the cohort (7.2%) required a cesarean due to arrest of descent or dilatation.

Our cohort included only women with spontaneous labor, as induction or augmentation after cesarean is used cautiously due to the risk of uterine rupture and typically involves reduced oxytocin dosing. This may limit generalizability, although previous studies reported no significant differences in labor progression between induced VBAC and spontaneous labor in primiparous women [13,16].

## 5. Conclusions

In this large cohort, women undergoing their first VBAC demonstrated a significantly faster cervical dilatation rate during the active phase and a shorter second stage of labor compared with primiparous women. VBAC was also inversely associated with prolonged second stage. These findings support the safety and efficiency of VBAC and may assist clinicians in counseling patients considering a trial of labor after cesarean. Further prospective studies are warranted to confirm these results and explore additional factors influencing labor progression.

These data may support more informed counseling of women who are candidates for TOLAC and contribute to reducing unnecessary repeat cesarean deliveries when VBAC is appropriate.

## Figures and Tables

**Table 1:** Clinical Characteristics of primiparous vs. secundiparous women who underwent VBAC.

	Primipara N = 13,983	VBAC N = 736	*p* Value
Age (years)	29.71 (±4.2)	32.6 (±3.6)	**<0.001**
BMI (kg/m^2^)	26.6 (±3.6)	26.9 (±3.9)	0.118
Height (cm)	164.7 (±6.1)	164.0 (±5.9)	0.596
Weight before pregnancy (kg)	58.92 (±9.6)	59.6 (±10.8)	0.09
Drugs Use	110 (0.8%)	8 (1.1%)	0.377
Smoking	577 (4.1%)	34 (4.6%)	0.528
Alcohol	38 (0.27%)	1 (0.13%)	0.262
Caucasian ethnicity	13,303 (95.1%)	713 (96.9%)	0.163
Asian ethnicity	7 (1%)	162 (1.2%)	0.163
African ethnicity	294 (2.1%)	10 (1.4%)	0.163

BMI = Body Mass Index; VBAC = Vaginal Birth After Cesarean. Values in bold indicate statistical significance (*p* < 0.05).

**Table 2:** Intrapartum and neonatal characteristics of primiparous vs. secundiparous women who underwent VBAC.

	Primipara N = 13,983	VBAC N = 736	*p* Value
Gestational Age (weeks)	39.56 (±1.07)	39.63 (±1.0)	0.144
Epidural Analgesia	10,987 (78.6%)	634 (86.1%)	**<0.001**
Second Stage (min)	86.6 (±59.9)	77.8 (±57.5)	**<0.001**
Prolonged Second Stage	1324 (9.5%)	52 (7.1%)	**0.029**
Cervical Dilatation Rate * (cm/h)	2.85 (±3.35)	3.261 (±4.91)	**0.011**
Intrapartum Fever	120 (0.86%)	7 (0.95%)	0.791
Episiotomy	713 (5.10%)	34 (4.70%)	0.259
OASI	85 (0.61%)	6 (0.81%)	0.524
Third Stage (min)	15.4 (±10)	15.1 (±9.6)	0.386
Hemoglobin Before Delivery (g/dL)	12.4 (±1.2)	12.4 (±1)	0.837
Hemoglobin After Delivery (g/dL)	11.6 (±1.7)	11.8 (±1.6)	0.053
Birthweight (g)	3234 (±378)	3266 (±367)	**0.025**
Male Gender	6950 (49.70%)	393 (53.40%)	0.051

* The cervical dilatation rate during the active phase of the first stage of labor. OASI = Obstetric Anal Sphincter Injury. Values in bold indicate statistical significance.

**Table 3:** Multivariable logistic regression analysis of factors associated with prolonged second stage (PSS).

	OR for PSS	95% CI	*p* Value
VBAC	0.541	0.388–0.753	**<0.001**
Maternal Age (years)	1.055	1.039–1.071	**<0.001**
BMI (kg/m^2^)	0.996	0.979–1.012	0.61
Cervical Dilatation Rate (cm/h)	0.805	0.771–0.841	**<0.001**
Epidural Analgesia	1.091	0.311–28.525	0.344
Caucasian Ethnicity	1.828	1.101–3.037	**0.02**
Asian Ethnicity	0.532	0.214–1.319	0.173
African Ethnicity	0.611	0.338–1.105	0.103
Neonatal Birth Weight (per each 1 kg)	1.88	1.602–2.204	**<0.001**

OR = Odds Ratio; CI = Confidence Interval; BMI = Body Mass Index; VBAC = Vaginal Birth After Cesarean; PSS = Prolonged Second Stage. Values in bold indicate statistical significance (*p* < 0.05).

## Data Availability

The data supporting the findings of this study are available from the corresponding author upon reasonable request.

## Abbreviations

The following abbreviations are used in this manuscript:

VBAC	Vaginal Birth After Cesarean
TOLAC	Trial of Labor After Cesarean
PSS	Prolonged Second Stage
BMI	Body Mass Index

## References

[1] Betrán A.P., Ye J., Moller A.B., Zhang J., Gülmezoglu A.M., Torloni M.R. (2016). The Increasing Trend in Caesarean Section Rates: Global, Regional and National Estimates: 1990–2014. PLoS ONE.

[2] Zhang J., Troendle J., Reddy U.M., Laughon S.K., Branch D.W., Burkman R., Landy H.J., Hibbard J.U., Haberman S., Ramirez M.M. (2010). Contemporary cesarean delivery practice in the United States. Am. J. Obstet. Gynecol..

[3] Sentilhes L., Vayssière C., Beucher G., Deneux-Tharaux C., Deruelle P., Diemunsch P., Gallot D., Haumonté J.-B., Heimann S., Kayem G. (2013). Delivery for women with a previous cesarean: Guidelines for clinical practice from the French College of Gynecologists and Obstetricians (CNGOF). Eur. J. Obstet. Gynecol. Reprod. Biol..

[4] Betrán A.P., Temmerman M., Kingdon C., Mohiddin A., Opiyo N., Torloni M.R., Zhang J., Musana O., Wanyonyi S.Z., Gülmezoglu A.M. (2018). Interventions to reduce unnecessary caesarean sections in healthy women and babies. Lancet.

[5] National Institutes of Health Consensus Development Conference Panel (2010). National Institutes of Health Consensus Development conference statement: Vaginal birth after cesarean: New insights March 8–10, 2010. Obstet. Gynecol..

[6] WHO (2018). WHO Recommendations: Intrapartum Care for a Positive Childbirth Experience.

[7] Ayres-de-Campos D., Simon A., Modi N., Tudose M., Saliba E., Wielgos M., Reyns M., Athanasiadis A., Stenback P., Verlohren S. (2024). European Association of Perinatal Medicine (Eapm) European Midwives Association (EMA). Joint position statement: Caesarean delivery rates at a country level should be in the 15–20% range. Eur. J. Obstet. Gynecol. Reprod. Biol..

[8] American College of Obstetricians and Gynecologists (2019). ACOG Practice bulletin no. 205: Vaginal Birth After Cesarean Delivery. Obstet. Gynecol..

[9] Gimovsky A.C. (2022). Evidence-based labor management: Second stage of labor (part 4). Am. J. Obstet. Gynecol..

[10] Barnea E.R., Ramasauskaite D., Ubom A.E., Di Simone N., Mueller M., Borovac-Pinheiro A., Guarano A., Benedetto C., Beyeza-Kashesya J., Nunes I. (2025). FIGO good practice recommendations for vaginal birth after cesarean section. Int. J. Gynaecol. Obstet..

[11] Houri O., Bercovich O., Berezovsky A., Gruber S.D., Pardo A., Werthimer A., Walfisch A., Hadar E. (2025). Success Rate and Obstetric Outcomes of Trial of Labor After Cesarean Delivery-Decision-Tree Analysis. Int. J. Gynaecol. Obstet..

[12] Shalev-Ram H., Miller N., David L., Issakov G., Weinberger H., Biron-Shental T. (2022). Spontaneous labor patterns among women attempting vaginal birth after cesarean delivery. Matern. Fetal Neonatal Med..

[13] Graseck A.S., Odibo A.O., Tuuli M., Roehl K.A., Macones G.A., Cahill A.G. (2012). Normal first stage of labor in women undergoing trial of labor after cesarean delivery. Obstet. Gynecol..

[14] Chazotte C., Madden R., Cohen W.R. (1990). Labor patterns in women with previous cesareans. Obstet. Gynecol..

[15] Karampas G., Witkowski M., Metallinou D., Steinwall M., Matsas A., Panoskaltsis T., Christopoulos P. (2023). Delivery Progress, Labor Interventions and Perinatal Outcome in Spontaneous Vaginal Delivery of Singleton Pregnancies between Nulliparous and Primiparous Women with One Previous Elective Cesarean Section: A Retrospective Comparative Study. Life.

[16] Grantz K.L., Gonzalez-Quintero V., Troendle J., Reddy U.M., Hinkle S.N., Kominiarek M.A., Lu Z., Zhang J. (2015). patterns in women attempting vaginal birth after cesarean with normal neonatal outcomes. Am. J. Obstet. Gynecol..

[17] Lan Y., Pan S., Chen B., Peng L., Chen R., Hua Y., Ma Y. (2022). Labor characteristics and intrapartum interventions in women with vaginal birth after cesarean section. BMC Pregnancy Childbirth.

[18] Li H., Yang L., Peng J., Cheng W., Ma H., Wu S., Wen J., Zhao Y. (2024). Duration time of labor progression for pregnant women of vaginal birth after cesarean in Hubei, China. Ir. J. Med. Sci..

[19] Laughon S.K., Branch D.W., Beaver J., Zhang J. (2012). Changes in labor patterns over 50 years. Am. J. Obstet. Gynecol..

[20] Cheng Y.W., Shaffer B.L., Nicholson J.M., Caughey A.B. (2014). Second stage of labor and epidural use: A larger effect than previously suggested. Obstet. Gynecol..

[21] ACOG Committee on Obstetric Practice (2002). ACOG Committee Opinion number 269 February 2002. Analgesia and cesarean delivery rates. American College of Obstetricians and Gynecologist. Obstet. Gynecol..

[22] Miller N., Pellegg M., Hag-Yahia N., Daykan Y., Pasternak Y., Biron-Shental T. (2019). Labor progression of women attempting vaginal birth after previous cesarean delivery with or without epidural analgesia. Arch. Gynecol. Obstet..

[23] Zhang J., Landy H.J., Branch D.W., Burkman R., Haberman S., Gregory K.D., Hatjis C.G., Ramirez M.M., Bailit J.L., Gonzalez-Quintero V.H. (2010). Contemporary patterns of spontaneous labor with normal neonatal outcomes. Obstet. Gynecol..

[24] Arulkumaran S., Gibb D.M.F., Lun K.C., Heng S.H., Ratnam S.S. (1984). The effect of parity on uterine activity in labour. Br. J. Obstet. Gynaecol..

[25] Friedman E.A. (1955). Primigravid labor: A graphicostatistical analysis. Obstet. Gynecol..

[26] Suzuki R., Horiuchi S., Ohtsu H. (2010). Evaluation of the labor curve in nulliparous Japanese women. Am. J. Obstet. Gynecol..

[27] Ashwal E., Livne M.Y., Benichou J.I.C., Unger R., Hiersch L., Aviram A., Mani A., Yogev Y. (2020). Contemporary patterns of labor in nulliparous and multiparous women. Am. J. Obstet. Gynecol..

[28] Harlass F.E., Duff P. (1990). The duration of labor in primiparas undergoing vaginal birth after cesarean delivery. Obstet. Gynecol..

[29] Faranesh R., Salim R. (2011). Labor progress among women attempting a trial of labor after cesarean. Do they have their own rules?. Acta Obstet. Gynecol. Scand..

[30] Rottenstreich M., Shahar C.F., Rotem R., Sela H.Y., Rottenstreich A., Samueloff A., Shen O., Reichman O. (2020). Duration of first vaginal birth following cesarean: Is stage of labor at previous cesarean a factor?. Eur. J. Obstet. Gynecol. Reprod. Biol..

[31] Rusavy Z., Francova E., Paymova L., Ismail K.M., Kalis V. (2019). Timing of cesarean and its impact on labor duration and genital tract trauma at the first subsequent vaginal birth: A retrospective cohort study. BMC Pregnancy Childbirth.

